# Effect of verapamil on the pharmacokinetics of hydroxycamptothecin and its potential mechanism

**DOI:** 10.1080/13880209.2020.1717550

**Published:** 2020-01-28

**Authors:** Hua Xing, Xiao Luo, Yang Li, Chunni Fan, Ning Liu, Chunguo Cui, Wenjia Li

**Affiliations:** Department of Breast Surgery, China–Japan Union Hospital of Jilin University, Changchun, China

**Keywords:** Drug–drug interaction, CYP3A4, P-gp

## Abstract

**Context:**

Hydroxycamptothecin (HCPT) has antitumor activity in various cancers, but its poor bioavailability and efflux limit its clinical application. Verapamil has been demonstrated to improve the bioavailability of many drugs. However, the effect of verapamil on the pharmacokinetics of HCPT was not clear.

**Objective:**

The effect of verapamil on the pharmacokinetics of HCPT was investigated to clarify the drug–drug interaction between HCPT and verapamil.

**Materials and methods:**

The pharmacokinetic profiles of oral administration of HCPT (50 mg/kg) in two group of Sprague–Dawley rats (six rats each), with pre-treatment of verapamil (10 mg/kg/day) for 7 days were investigated, with the group without verapamil pre-treatment as control. Additionally, the metabolic stability and transport of HCPT in the presence or absence of verapamil were also investigated with the employment of the rat liver microsomes and Caco-2 cell transwell model.

**Results:**

Verapamil significantly increased the peak plasma concentration (from 91.97 ± 11.30 to 125.30 ± 13.50 ng/mL), and decrease the oral clearance (from 63.85 ± 10.79 to 32.95 ± 6.17 L/h/kg). The intrinsic clearance rate was also significantly decreased (from 39.49 ± 0.42 to 28.64 ± 0.30 μL/min/mg protein) by the preincubation of verapamil. The results of Caco-2 cell transwell experiments showed the efflux of HCPT was inhibited by verapamil, as the efflux ratio decreased from 1.82 to 1.21.

**Discussion and conclusions:**

The system exposure of HCPT was increased by verapamil. Verapamil may exert this effect through inhibiting the activity of CYP3A4 or *P-gp*, which are related to the metabolism and transport of HCPT.

## Introduction

Hydroxycamptothecin (HCPT) is one of the analogues of camptothecin that isolated from the Chinese tree *Camptotheca acuminate* Decne (Nyssaceae) (Wall et al. 1966). HCPT can inhibit DNA replication and RNA synthesis, which leads to antitumor activity (Khokhlov 1976; Yoshida et al. 1993). For example, HCPT can mediate the apoptosis of cervical cancer via autophagy activation (Cheng et al. 2016). HCPT has been employed for the treatment of a broad spectrum of cancers in China (Pu et al. 2009), including gastric carcinoma, hepatoma, leukaemia, and tumours of head and neck (Zheng et al. 2012). However, the bioavailability of HCPT was not ideal (Zhang et al. 2004), which limited its application. Previous studies reported the combination of HCPT and other drugs can improve the antitumor ability of HCPT in human oral squamous cell carcinoma and breast cancer cells (Jiang et al. 2010; Ge et al. 2019). Therefore, the bioavailability of HCPT may be enhanced when it co-administrated with other drugs.

Verapamil is a kind of calcium channel blocker. Verapamil is also a specific inhibitor of *P-gp* and CYP3A4, which are closely associated with the transport and metabolism of a number of drugs (Srinivas 2008). Previous studies have reported many drugs, of which the pharmacokinetics can be affected when combined with verapamil. For instance, the *C*_max_ and *AUC*_(0–t)_ of sorafenib were increased when co-administered with verapamil (Wang et al. 2016). The coadministration of verapamil and ibrutinib can exert severe toxicity due to the drug-drug interaction (Lambert et al. 2016). Many other drugs that may be coadminstrated with verapamil in the clinic, were also reported to be influenced by verapamil, such as dihydromyricetin, puerarin, and paclitaxel (Choi and Li 2005; Huang et al. 2018; Zhou et al. 2019). In addition, HCPT is a substrate of *P-gp* and its metabolism is mainly mediated by CYP3A4 (Pu et al. 2011; Burney et al. 2017). Therefore, the drug–drug interaction between HCPT and verapamil may occur, and it is necessary to investigate the effect of verapamil on the pharmacokinetics of HCPT, which directly affects the bioavailability of HCPT.

The effect of verapamil on the pharmacokinetics of HCPT was investigated in this research, to explore the interaction between HCPT and verapamil, which can provide information relevant to the clinical application of HCPT. The *in vivo* pharmacokinetics of HCPT in rats with or without verapamil pre-treatment were determined using a sensitive LC-MS/MS method. Additionally, the effects of verapamil on the metabolism stability of HCPT were investigated with rat liver microsomes and the Caco-2 cell transwell model.

## Material and methods

### Chemicals

Verapamil (purity > 98%) and HCPT (purity > 98%) were obtained from Shanghai Standard Biotechnology Co., Ltd (Shanghai, China). Acetonitrile and methanol were purchased from Fisher Scientific (Fair Lawn, NJ). Dulbecco’s modified Eagle’s medium (DMEM) and non-essential amino acid (NEAA) solution were purchased from Thermo Scientific Corp. (Logan, UT). Foetal bovine serum (FBS) was obtained from GIBCO BRL (Grand Island, NY). Penicillin G (10,000 U/mL) and Streptomycin (10 mg/mL) were purchased from Amresco (Solon, OH). Hanks’ balanced salt solution (HBSS) was purchased from GIBCO (Grand Island, NY). Ultrapure water was prepared with a Milli-Q water purification system (Millipore, Billerica, MA). All other chemicals were of analytical grade or better.

### Animal experiments

Male Sprague–Dawley rats weighing 230–250 g were provided by Shanghai SLAC Laboratory Animal Co., Ltd (Shanghai, China). Rats were bred in a breeding room at 25 °C with 60 ± 5% humidity and a 12 h dark-light cycle. Tap water and normal chow were given *ad libitum*. All of the experimental animals were housed under the above conditions, for a 3-day acclimation period and fasted overnight before the experiments. All experimental procedures and protocols were reviewed and approved by the Animal Care and Use Committee of China–Japan Union Hospital of Jilin University and were in accordance with the National Institutes of Health guidelines regarding the principles of animal care.

### *In vivo* pharmacokinetic study

Rats were divided into two groups of six animals each, with or without pre-treatment of verapamil. The pre-treatment of verapamil on the test group was at a dose of 10 mg/kg/day (dissolved directly in normal saline containing 0.5% methylcellulose at a concentration of 2 mg/mL) for 7 days. Next, 50 mg/kg HCPT was administrated orally. Blood samples (200 μL) were collected into heparinized tubes via the oculi choroidal vein at 0.083, 0.33, 0.5, 1, 2, 4, 6, 8, 10, 12, 24, and 36 h after the oral administration of verapamil. The blood samples were centrifuged at 3500 rpm for 5 min. The plasma samples that were obtained were stored at −40 °C until analysis.

### Preparation of rat plasma samples

To obtain 100 μL aliquot of plasma sample, 20 μL methanol and 180 μL internal standard methanol solution (2 ng/mL) were added and vortexed for 60 s to mix in a 1.5 mL polypropylene tube, and were centrifuged at 12,000 rpm for 10 min. The supernatant was removed into an injection vial, and a 3 μL aliquot was injected into the LC-MS/MS system for analysis.

### LC-MS/MS determination of HCPT

The determination of HCPT was performed on an Agilent 1290 series liquid chromatography system and an Agilent 6470 triple-quadruple mass spectrometer (Palo Alto, CA). The HPLC/MS conditions and sample preparation were basically according to a validated HPLC method described before (Zhang et al. 2019). The chromatographic analysis of HCPT was performed on a Waters X-Bridge C18 column (3.0 × 100 mm, i.d.; 3.5 μm, Waters Corporation, Milford, MA ) at room temperature (25 °C). The mobile phase was water (containing 0.1% formic acid) and acetonitrile (30:70, v: v) with isocratic elution at a flow rate of 0.2 mL/min, and the analysis time was 4 min.

The mass scan mode was positive MRM mode. The precursor ion and product ion are *m*/*z* 365.20→321.10 for HCPT and *m*/*z* 349.25→305.15 for IS. The collision energy for HCPT and IS were 30 and 20 eV, respectively. The MS/MS conditions were optimized as follows: fragmentor, 110 V; capillary voltage, 3.5 kV; Nozzle voltage, 500 V; nebulizer gas pressure (N_2_), 40 psig; drying gas flow (N_2_), 10 L/min; gas temperature, 350 °C; sheath gas temperature, 400 °C; sheath gas flow, 11 L/min.

### Cell culture

The Caco-2 cell line was obtained from the American Type Culture Collection (Manassas, VA), and it was performed according to the previous study (Liu et al. 2019). The Caco-2 cells were cultured in DMEM high glucose medium containing 15% FBS, 1% NEAA, and 100 U/mL penicillin and streptomycin. The cells were cultured at 37 °C with 5% CO_2_. For transport studies, the cells at passage 40 were seeded on transwell polycarbonate insert filters (1.12 cm^2^ surface, 0.4 μm pore size, 12 mm diameter; Corning Co-star Corporation, Corning, MA) in 12-well plates at a density of 1 × 10^5^ cells/cm^2^. Cells were allowed to grow for 21 days. For the first seven days, the medium was replaced every two days, and then daily. The transepithelial electrical resistance (TEER) of the monolayer cells was measured using Millicell ERS-2 (Millipore Corporation, Billerica, MA), and TEER exceeding 400 Ω·cm^2^ was used for the flux experiment. The integrity of the Caco-2 monolayers was confirmed by the paracellular flux of Lucifer yellow, which was less than 1% per hour. The alkaline phosphatase activity was validated using an Alkaline Phosphatase Assay Kit. The qualified monolayers were used for transport studies.

### Effects of verapamil on the transport of HCPT in the Caco-2 cell transwell model

The Caco-2 cell transwell model was employed to investigate the transport of HCPT in the presence or absence of verapamil. The cell monolayers were rinsed twice with warm (37 °C) Hanks’ balanced salt solution (HBSS) before the transport experiments. After that, the cells were incubated at 37 °C for 20 min. HCPT was added to either apical or basolateral side to incubate with the cell monolayers in fresh incubation medium for the indicated times at 37 °C, after preincubation. The volume of incubation medium on the apical and basolateral sides was 0.5 mL and 1.5 mL, respectively, and a 100 μL aliquot of the incubation solution was withdrawn at the indicated time points from the receiver compartment and replaced with the same volume of fresh pre-warmed HBSS buffer. The permeability of HCPT (2 μM) in all of the above conditions for both directions, i.e., from the apical (AP) side to the basolateral (BL) side and from the BL side to the AP side, was measured after incubation for 30, 60, 90, and 120 min at 37 °C. In addition, the efflux activity of *P-gp* was validated using a typical *P-gp* substrate digoxin (25 μM).

The apparent permeability coefficient (*P_app_*) was calculated using the equation of Artursson and Karlsson:

$$P_{\mathrm{app}}=\left(\Delta Q/\Delta t\right)\times \left[1/\left(A\times C_{0}\right)\right]$$

where *P_app_* is the apparent permeability coefficient (cm/s), Δ*Q*/Δ*t* (μmol/s) is the rate at which the compound appears in the receiver chamber, *C*_0_ (μmol/L) is the initial concentration of the compound in the donor chamber, and *A* (cm^2^) represents the surface area of the cell monolayer. Data were collected from three separate experiments, and each was performed in triplicate.

### Effects of verapamil on the metabolic stability of HCPT in rat liver microsomes

The metabolic stability of HCPT was investigated in rat liver microsomes with or without verapamil. The detailed methods were similar to those reported before (Wang et al. 2017; Yan et al. 2017). Briefly, 30 μL rat liver microsome (20 mg/mL), 12 μL HCPT solution (100 μM) and 1113 μL PBS buffer (0.1 M, pH 7.4) were added to the centrifuge tubes on ice. After a 5-min preincubation at 37 °C, the NADPH-generating system (45 μL) was added into the microsomal suspension to initiate the reaction. Meanwhile, the effects of verapamil or ketoconazole (a positive CYP3A4 inhibitor) on the metabolic stability of HCPT were investigated by adding 10 μM of verapamil or ketoconazole (12 μL, final concentration of 0.1 μM) to rat liver microsomes and preincubating them for 30 min at 37 °C, followed by the addition of NADPH-generating system. Aliquots of 100 μL were collected from the reaction volumes at 0.083, 0.167, 0.33, 0.5, 1, 2, 4, 8, 12, 24, and 36 h after the addition of HCPT, and 200 μL ice-cold acetonitrile containing esculin was added to terminate the reaction. All the experiments were performed in triplicate. The subsequent sample preparation method was the same as the plasma sample preparation method, and the concentration of HCPT was determined by LC-MS.

The *in vitro* half-life (*t_1/2_*) was obtained using the equation: *t_1/2_ =* 0.693/*k*; V (μL/mg) = volume of incubation (μL)/protein in the incubation (mg); intrinsic clearance (Clint) (μL/min/mg protein) = V × 0.693/*t_1/2_*.

### Data analysis

Pharmacokinetic parameters, including the area under the plasma concentration–time curve (*AUC*), maximal plasma concentration (*C*_max_), the time for the maximal plasma concentration (*T*_max_), and the mean residence time (*MRT*), were calculated using the DAS 3.0 pharmacokinetic software (Chinese Pharmacological Association, Anhui, China).

The differences between the mean values were analyzed for significance using a one-way analysis of variance (ANOVA). Values of *p <* 0.05 were considered to be statistically significant.

## Results

### Effect of verapamil on the pharmacokinetics of HCPT

**Figure 1.**
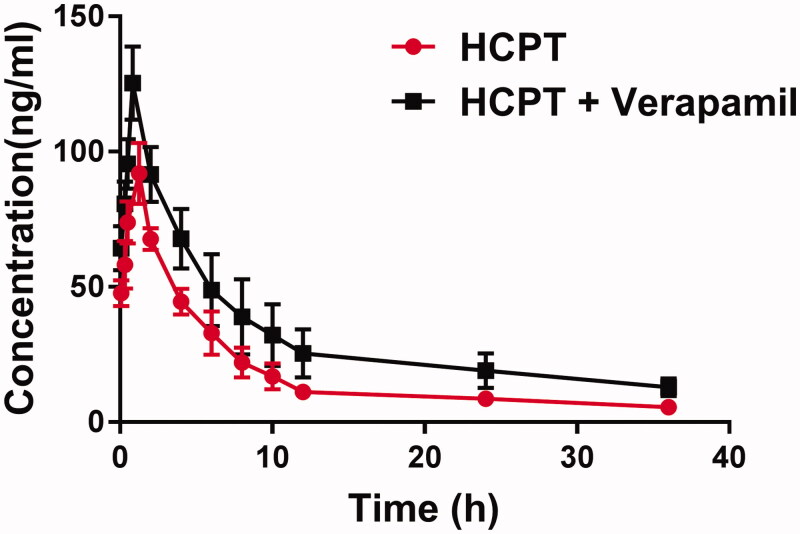
The pharmacokinetic profiles of HCPT in rats (six rats in each group) after the oral administration of 50 mg/kg HCPT with or without verapamil pre-treatment (10 mg/kg/day for 7 days). Each point represents the average ± S.D. of six determinations.

The mean plasma concentration-time curves of HCPT in the presence or absence of verapamil are shown in Figure 1. Meanwhile, the pharmacokinetic parameters were calculated by the noncompartmental method with the DAS 3.0 pharmacokinetic software (Chinese Pharmacological Association, Anhui, China) and summarized in Table 1.

**Table 1.** Pharmacokinetic parameters of pinocembrin in rats after intragastrical administration of HCPT (50 mg/kg; *n* = 6, Mean ± S.D.) with or without treatment of verapamil.

Parameter	Control	Pre-treatment of verapamil
*T*_max_ (h)	1.12 ± 0.14	0.89 ± 0.09
*C*_max_ (ng/mL)	91.97 ± 11.30	125.30 ± 13.50*
*t*_1/2_ (h)	17.71 ± 3.60	23.00 ± 6.71*
AUC _(0–t)_ (mg·h/L)	0.66 ± 0.10	1.14 ± 0.29*
MRT (h)	9.61 ± 0.55	11.45 ± 0.78*
CLz/F (L/h/kg)	63.85 ± 10.79	32.95 ± 6.17*

*p < 0.05 indicate significant differences from the control.

The administration of verapamil significantly increased the peak plasma concentration (*C*_max_) of HCPT (125.30 ± 13.50 versus 91.97 ± 11.30 ng/mL, *p* < 0.05). The *AUC_0–t_* of HCPT was also significantly increased from 0.66 ± 0.10 to 1.14 ± 0.29 mg h/L in the presence of verapamil (*p* < 0.05). These results indicated verapamil can improve the concentration of HCPT in plasma. In addition, verapamil significantly prolonged the half-life (*t_1/2_*) and the mean residence time (MRT) of HCPT and inhibited the clearance rate (*p* < 0.05), which indicated the system exposure of HCPT was increased by the preincubation of verapamil.

### Effect of verapamil on the metabolic stability of HCPT in rat liver microsomes

In rate liver microsomes, the metabolic half-life of HCPT was 35.1 ± 0.32 min, and it prolonged to 48.4 ± 0.51 min in the presence of verapamil; the difference was significant (*p* < 0.05). Moreover, the intrinsic clearance rate was reduced from 39.49 ± 0.42 to 28.64 ± 0.30 μL/min/mg protein by verapamil. The prolonged metabolic half-life and the reduced intrinsic clearance rate verified the results of pharmacokinetics experiments. Both results also suggested the metabolic stability of HCPT in rat liver microsomes was enhanced by verapamil.

### Effect of verapamil on the bidirectional transport of HCPT in Caco-2 cell transwell mode

**Figure 2.**
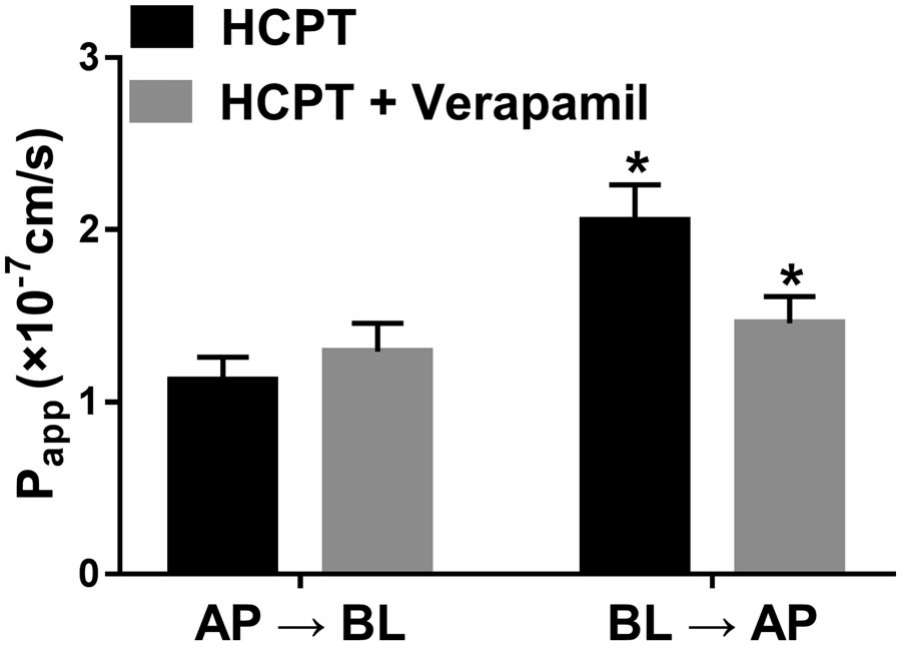
Effects of verapamil on the transport of HCPT from the apical to basolateral side or the opposite direction, Caco-2 cell monolayers were incubated at 37 °C in HBSS (pH 7.4), and HCPT (2 µM) were added to the apical or basolateral side, verapamil were also added to the donor chamber with HCPT. *Significant differences (*p* < 0.05) were seen compared to the control sample. Each point represents the mean ± SD of 3 determinations.

The transport of HCPT with or without pre-treatment of verapamil was investigated in the Caco-2 cell transwell model. First, to validate the efflux activity of *P-gp*, a specific *P-gp* substrate, digoxin was employed. The results showed the efflux ratio of digoxin was 11.03, and the addition of verapamil abrogated it, which suggested the qualified activity of *P-gp*. Figure 2 shows the apparent permeability coefficient (*P_app_*) of HCPT from the AP side to the BL side (*Papp_AB_*) and from the BL side to the AP side (*Papp_BA_*). The *Papp_BA_* of HCPT was significantly higher than *Papp_AB_* with the efflux ratio of 1.82. In the presence of verapamil, the *Papp_BA_* of HCPT was significantly decreased from 2.05 ± 0.21 × 10^−7^ to 1.46 ± 0.16 × 10^−7 ^cm/s, as the efflux ratio decreased to 1.21. These results indicated that verapamil inhibited the efflux of HCPT.

## Discussion

HCPT is a naturally occurring alkaloid anticancer agent in China, which has demonstrated antitumor activity towards a wide range of cancer, such as lung cancer, colorectal cancer, breast cancer, and cervical cancer (Zaki 2014; Cheng et al. 2016; Ge et al. 2019). In Chinese clinics, HCPT has been applied in the treatment for some cancers including gastric carcinoma, hepatoma, leukaemia, and tumours of head and neck (Zheng et al. 2012). However, the poor bioavailability and stability limited its clinical use (Hofheinz et al. 2005). Previous studies have reported the co-administration of HCPT and with other drugs can improve the bioavailability and pharmacodynamic effect of HCPT. For example, the antitumor effect of HCPT on bladder cancer can be enhanced when it combined with triptolide (Wang et al. 2019). Paris Saponin I can improve HCPT to induce the apoptosis of lung cancer cells (Liu et al. 2017). Verapamil is a specific inhibitor of *P-gp* and CYP3A4, on which the transport and metabolism of most drugs are dependent (Srinivas 2008). Many drugs with poor oral bioavailability co-administrated with verapamil could improve their absorption (Choi and Li 2005; Huang et al. 2018; Zhou et al. 2019). Therefore, the interaction between HCPT and verapamil was studied in this paper to assess the effect of verapamil on the pharmacokinetics of HCPT.

In the present study, it was found that the system exposure of HCPT was increased by the administration of verapamil, as the value of *C*_max_ and *AUC_(0_*_–_*_t)_* increased and the *t_1/2_* and oral clearance of HCPT decreased. In rat liver microsomes, the metabolic half-life and intrinsic clearance rate of HCPT were also inhibited by verapamil. These results indicated the inhibitory effect of verapamil on the metabolism of HCPT. HCPT has been demonstrated to be metabolized by CYP3A4, of which the activity can be inhibited by verapamil (Srinivas 2008; Burney et al. 2017). Therefore, we inferred that verapamil affected the pharmacokinetics of HCPT via inhibiting the activity of CYP3A4.

From the results of Caco-2 cell transwell mode, we found the transporter *P-gp* was involved in the transport of HCPT, as the efflux of HCPT was much higher than the influx, which is consistent with previous studies (Pu et al. 2009; Pu et al. 2011). The results showed that verapamil inhibited the efflux of HCPT and promoted the absorption of HCPT. Verapamil has been considered as a specific inhibitor of *P-gp*, and it also inhibited the transport of many kinds of drugs via inhibiting the activity of *P-gp* (Bansal et al. 2009; Alvariza et al. 2013; Huang et al. 2018; Zhou et al. 2019). Therefore, it can be speculated that verapamil inhibited the transport of HCPT through affecting the activity of *P-gp*.

Based on the above, the metabolism and transport of HCPT can be inhibited by verapamil when they are co-administrated. Verapamil inhibited the metabolism of HCPT by inhibiting the activity of CYP3A4, and inhibiting its transport by inhibiting the activity of *P-gp*. However, the poor solubility and dissolution rate are also two important factors that affect the bioavailability and pharmacodynamics effect of HCPT. The roles of other metabolism enzymes or other transporters might be involved during the drug-drug interaction. These results may be further defined in future studies.

## Conclusions

The co-administration of HCPT and verapamil can significantly influence the pharmacokinetic profile of HCPT. The system exposure of HCPT was increased by verapamil, which may result from the inhibition of CYP3A4 and the inhibition of *P-gp*.
